# Application of Intraoperative Radiotherapy in Early-Stage Breast Cancer Patients in a Clinical Setting

**DOI:** 10.1155/2024/5551907

**Published:** 2024-06-18

**Authors:** Ao Wang, Elchanan Quint, Ivan Kukeev, Ravit Agassi, Olga Belochitski, Gay Barski, Julie Vaynshtein

**Affiliations:** ^1^ Medical School for International Health Ben-Gurion University of the Negev, Be'er Sheva 8410501, Israel; ^2^ Department of General Surgery B Soroka University Medical Center, Be'er Sheva 8410101, Israel; ^3^ Center Surgical Oncology of Breast Soroka University Medical Center, Be'er Sheva 8410101, Israel; ^4^ Radiation Oncology Unit Soroka University Medical Center, Be'er Sheva, Israel

## Abstract

**Background:** Intraoperative radiation therapy (IORT) has gained popularity in recent years as an alternative to external beam whole breast radiation therapy (WBRT) for early-stage breast cancer. Here, we report 43-month recurrence and survival outcomes in a multiethnic cohort treated with IORT in a clinical context.

**Method:** Two hundred and eleven patients with low-risk features were treated with IORT for early-stage breast cancer from 2014 to 2021. Selection criteria were based on Group Europeen de Curietherapie-European Society for Therapeutic Radiology and Oncology (GEC-ESTRO) guidelines: preferably unifocal intraductal carcinoma (IDC), aged > 50, tumor size ≤ 2.0 cm, and without lymph node involvement. All patients received 20 Gy of radiation dose during the lumpectomy. Information on patient and tumor characteristics was collected.

**Results:** The mean age of this cohort was 67.5 years; 95.2% of patients are Jewish, and the rest are Bedouins (4.7%). Most tumors were intraductal carcinoma (97.2%) and stage 1 (94.8%). The mean follow-up time was 43.4 months. Bedouins had larger tumor sizes (mean 1.21 vs. 1.13 cm) and were younger at diagnosis than Jewish patients (mean 65.4 vs. 67.6 years), although the differences are not significant. The overall recurrence rate was 1.4%. One case of local recurrence (0.5%) and two cases of metastasis (0.9%) were observed during the study period. One patient died from metastasis.

**Conclusion:** Our findings suggest that IORT in selected low-risk patients can achieve an excellent prognosis with low rates of recurrence and metastasis.

## 1. Introduction

As of 2020, breast cancer has overtaken lung cancer to become the most common cancer globally, accounting for 1 in 4 cancer cases and 1 in 6 cancer deaths in women [1]. Breast cancer is also the leading cause of cancer-related death among females in both developed and developing countries [2]. For many patients with early-stage breast cancer, breast-conserving surgery (BCS) with adjuvant radiotherapy has equivalent long-term survival compared with radical mastectomy [3]. Traditionally, whole breast radiation therapy (WBRT) is the standard of practice following BCS to reduce local recurrence and improve survival [4, 5]. However, WBRT requires daily radiation for up to 7 weeks, which is a significant burden on many patients (1.8–2.0 Gy/fraction, 25–33 fractions). Such a prolonged postoperative radiation regimen is associated with noncompliance, and missed treatment can potentially increase the risk of cancer recurrence or death [6–8]. Intraoperative radiation therapy (IORT) is a new alternative to WBRT that delivers a single dose of 20 Gy radiation during lumpectomy to the tumor bed. By striking the target area directly, IORT avoids damage to healthy tissues and reduces treatment-associated adverse effects such as tissue fibrosis and necrosis [9, 10]. In addition, IORT resolves the issue of noncompliance since there is no longer a need for daily travel to the treatment center. Results from the TARGIT-A trial showed that in patients with early-stage breast cancer, BCS with IORT had similar local disease recurrence and cancer survival outcomes compared to WBRT after 30 months [11]. The follow-up study of the original TARGIT-A trial showed that in patients who received risk-adapted IORT, there was no difference in local tumor recurrence rate or overall survival after 8.6 years compared to those treated with WBRT [12]. However, another landmark study with a median follow-up of 5.8 years found that IORT had more ipsilateral breast tumor recurrence compared to WBRT, although overall survival was not different between the two modalities [13].

Although a growing number of studies have demonstrated promising results from utilizing IORT in early-stage breast cancer, whether IORT is a safe long-term alternative to WBRT remains unclear. This lack of confidence came from several randomized studies that failed to consistently demonstrate the noninferiority of IORT in local disease control compared to WBRT [14–16]. Additional long-term data are urgently needed to evaluate the efficacy of IORT and to identify patient characteristics suitable for this treatment modality. Nonetheless, IORT has practical utility in clinical settings where access to daily postoperative radiotherapy is difficult. This is especially true for patients from disadvantaged backgrounds. Here, we report recurrence and cancer survival outcomes in patients with early-stage breast cancer treated with IORT at the Soroka Medical Center Breast Surgery Unit. Patients were residents of the Negev area, a periphery and underdeveloped region in Israel. Our aim is to determine if IORT can achieve good clinical outcomes in suitable patients and to assess immediate treatment-associated side effects.

## 2. Methods

### 2.1. Eligibility Criteria

From 2014 to 2021, eligible patients with early-stage breast cancer were treated with IORT based on the recommendations of the Group Europeen de Curietherapie-European Society for Therapeutic Radiology and Oncology (GEC-ESTRO) guideline [17]. The selection criteria included unifocal, aged > 50, preferably with a tumor size ≤ 3.0 cm, and without lymph node involvement. All patients received 20 Gy of radiation dose during the lumpectomy. If the final pathology revealed malignant cells inside the tumor margin or micrometastasis in axillary lymph nodes, then patients were treated with adjuvant WBRT.

The data in this study were retrospectively collected from our hospital's electronic medical record system. The data collection was performed by manual surveillance of the patient's computerized files. We gathered information on patient demographics, tumor characteristics, adjuvant therapy, continuity of treatment, follow-up duration, tumor recurrence, and survival outcome. Treatment-associated toxicity was evaluated using late effects in normal tissues—subjective, objective, management, and analytic criteria (LENT-SOMA). This study was approved by the institutional Helsinki board.

### 2.2. Statistical Analysis

All statistical analysis in this study was done with RStudio 3.3.0+. Continuous variables such as age and tumor size were summarized using the mean and standard deviation. Categorical variables were summarized using percentages. Because of the considerable difference in sample size between Jewish and Bedouin patients, the Welch test was used to compare continuous variables (i.e., age and tumor size) between groups. Fisher's exact test was used to compare the frequency of categorical variables (i.e., recurrence and death rate) between groups. Disease-free survival (DFS) was defined as the time from diagnosis to the time of any tumor recurrence or death. The Kaplan–Meier survival curve was drawn using the Survival package in R. Logistic regression was used in the multivariate analysis.

## 3. Results

### 3.1. Demographics and Tumor Characteristics

**Figure 1:**
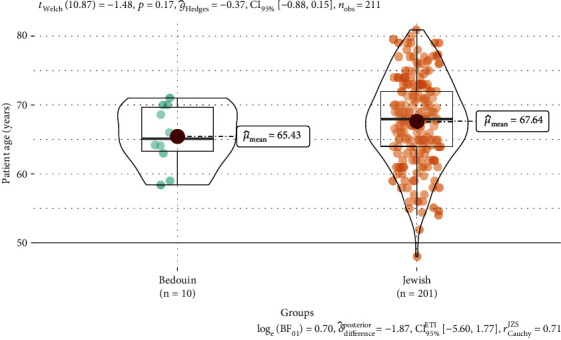
Violin plot of age at diagnosis by ethnic group. The solid line inside the box represents the mean which is also shown by the numeric value. The vertical line outside the box represents data within the 1.5 interquartile range. The shape of the figure represents the distribution of data points along the *y*-axis.

**Figure 2:**
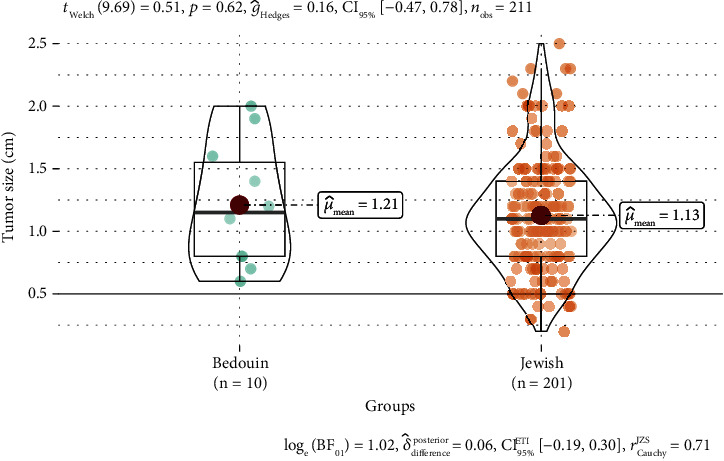
Violin plot of tumor size by ethnic group. The solid line inside the box represents the mean which is also shown by the numeric value. The vertical line outside the box represents data within the 1.5 interquartile range. The shape of the figure represents the distribution of data points along the *y*-axis.

The total number of patients included in this study from the electronic record system was 211, of which 201 were Jewish and 10 were Bedouin. Patient and tumor characteristics are summarized in Table 1. One hundred and twelve patients had left-sided cancer, while ninety-six patients had right-sided cancer. Three patients had bilateral cancer. The mean age of the whole cohort was 67.5 years (range: 48–80.9 years). Jewish patients were older at diagnosis than Bedouin patients (mean 67.6 vs. 65.4 years, *p* = 0.17, Figure 1). On the other hand, Bedouin patients had a larger tumor size compared to Jews (mean 1.21 vs. 1.13 cm, *p* = 0.62, Figure 2). Most tumors were intraductal carcinoma (IDC, 97.2%) and the rest were intralobular carcinoma (ILC, 1.4%) or mucinous carcinoma (1.4%). Jews had a higher ratio of IDC carcinoma compared to Bedouins (98.5% vs. 70%, *p* = 0.001). Most tumors in both groups are stage 1 (95% in Jews and 99% in Bedouin). Postpathology lymph node metastasis was found in 8.5% of Jewish patients and 10% of Bedouin patients. All tumors were ER-positive (100%), and most tumors were also positive for progesterone (94.3%). Most patients in both groups received adjuvant hormonal therapy (Jews 97.5% vs. Bedouin 90%, *p* = 0.26). There were 11 Jewish patients with confirmed HER2-positive tumors. Fifty-seven (28.4%) Jewish patients and four (40%) Bedouin patients had ONCOTYPE recurrence scores on the primary tumor. Eleven patients had an ONCOTYPE score greater than 25 which indicates a significant survival benefit from additional chemotherapy. Seven patients opted to receive additional chemotherapy.

### 3.2. Treatment Outcome

**Figure 3:**
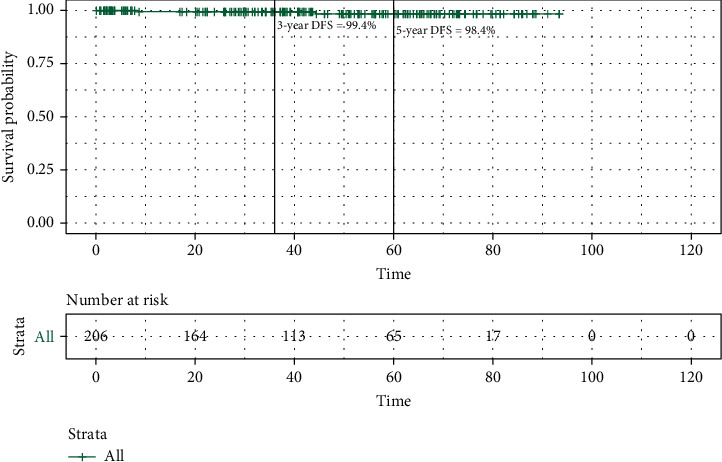
Disease-free survival (DFS) curve of the whole cohort. The vertical axis shows the percentage of survival, and the horizontal axis shows the follow-up time in months. Small vertical lines on the curve represent censored patients. At 3 years after initial surgery, the DFS is 99.4%. At 5 years, the DFS is 98.4%.

**Figure 4:**
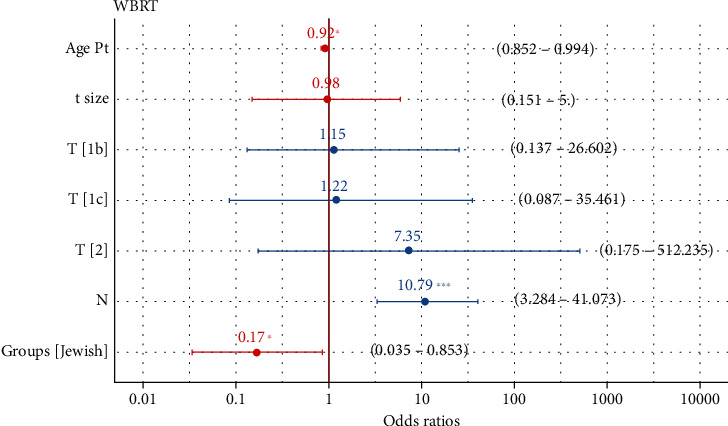
Multivariate analysis of factors associated with the likelihood of postsurgery WBRT. Patient age, tumor size, T stage, lymph node involvement, and ethnic group are included. Variables are included either from a clinical perspective or because they are significant in univariate analysis. The odds ratio is shown on the *x*-axis. Numbers on the solid line for each variable indicate the exact odds ratio, while numbers in the bracket represent 95% confidence intervals. The asterisk indicates significant variables.

The median follow-up time was 43.4 months for this cohort (Table 2). There was no difference in follow-up duration between Jewish and Bedouin patients (mean 43.8 vs. 29.9 months, *p* = 0.46). The DFS rate was 99.4% at 3 years and 98.4% at 5 years. At the conclusion of this study, the median DFS was not reached (Figure 3). A subset of 27 patients received postoperative WBRT based on the pathological evaluation of the tumor which revealed high-risk features of recurrence. Those who underwent WBRT had larger tumors (median 1.2 vs. 1.05 cm, *p* < 0.001) and a younger age at diagnosis (66.6 vs. 68 years, *p* < 0.001). Notably, more Bedouin patients received WBRT compared to Jewish patients (40% vs. 11.4%, *p* = 0.03). The logistic multivariate model showed that age, N stage, and ethnicity were significantly associated with the likelihood of receiving adjuvant WBRT (Figure 4).

Overall, only one case of local cancer recurrence was observed in a Jewish patient. This patient was 70 years old at the time of diagnosis with a primary IDC of 2.2 cm. After 43 months of initial surgery, she had a recurrence of DCIS in the same breast. She underwent a second operation and has remained cancer-free since. Two cases of metastasis were observed. The first case was in a 67-year-old Jewish patient with a primary IDC of 1.1 cm. Metastatic IDC was found 9 months after the initial operation. She underwent reoperation with sentinel lymph node dissection and has remained cancer-free since. The second case was a 59-year-old Bedouin patient with a primary IDC of 1.2 cm. Metastasis was noted after 39 months of initial surgery, and she died of cancer at 56 months.

### 3.3. Treatment-Associated Adverse Effect

The most common treatment-related effects were fibrosis and mild local tissue pain (both occurring in less than 30% of patients). One case of skin infection was observed which is less frequent compared to other IORT studies [18, 19]. No grade 3 and above adverse effects such as lymphedema or telangiectasia were seen in any patients according to the LENT-SOMA grading scale for toxicity.

## 4. Discussion

The incidence of local recurrence following BCS has been reduced significantly over the last couple of decades due to the utilization of radiotherapy. In a large meta-analysis published in 2011, radiotherapy reduced the 10-year risk of recurrence in early-stage breast cancer from 31% to 15.6% and the 15-year risk of death from 20.5% to 17.2% [4]. With the advance of technology, new treatment modalities in radiation therapy have emerged, including IORT. In the first two landmark studies on IORT, the local recurrence rate was 2.1% in TARGIT-IORT and 4.4% in ELIOT. The cancer-related mortality rate was 5.7% in TARGIT-IORT and 2.1% in ELIOT [12, 13]. Although both trials showed excellent local disease control and survival rates in the initial follow-up period, there is still debate on the effectiveness of IORT as an alternative to traditional WBRT in the long run [14]. While there is a lack of other large-scale randomized trials comparing those two modalities, many smaller studies have shown promising outcomes of IORT in well-selected patients with early-stage breast cancer. One recent French study of 225 elderly women treated with partial mastectomy with IORT showed a local recurrence rate of 1.7% after 5 years [20]. Another Israeli study of 158 women showed excellent results, with no local recurrence after 30 months [21]. Other studies of varying sample sizes have reported local recurrence rates between 1% and 5.8% [22–26].

Most patients in the current study meet the selection criteria of GEC-ESTRO for partial breast irradiation: age greater than 50, primary tumor size smaller than 3 cm, and no lymph node infiltration [17]. Only one (0.5%) patient was younger than 50. Most patients had tumors less than 2 cm, and only seven (3.3%) patients had tumors between 2 and 2.5 cm. The final pathology examination of lymph nodes dissected during surgery revealed 18 (8.5%) patients had nodal micrometastasis. In the current study, both local recurrence rates and cancer-related mortality rates are 0.5%, which are superior to the outcomes achieved by the TARGIT-IORT and EILOT trials. Furthermore, no severe acute or chronic treatment-associated adverse effects were found in our patients. Only tissue fibrosis and mild pain were observed during follow-up. Those results demonstrated that IORT has low toxicity to normal tissues and good cosmetic outcomes, as reported by others [27–29]. Our findings suggest that IORT is a safe and feasible alternative to traditional WBRT in well-selected patients. Favorable characteristics for consideration of IORT include early tumor stage, small tumor size, and limited extrabreast involvement. Only a subset of patients (12.8%) in our study needed additional WBRT based on postoperative pathology evaluation of tumor and nodal infiltration. Multivariate analysis revealed that younger patient age, nodal involvement, and Bedouin ethnicity are associated with a higher likelihood of requiring additional WBRT. This could be explained by the observation that Bedouin patients were younger and had larger tumors than Jewish patients, which may necessitate more aggressive treatment. However, studies with larger sample sizes are needed to fully assess factors associated with additional WBRT after the initial IORT.

The present study is the first report describing the outcome of women with early-stage breast cancer treated with IORT in the Negev, a unique region in southern Israel. It is the largest province in the country by land mass, but it also has the lowest average income in the nation. Soroka University Medical Center is a tertiary hospital that serves more than one million population in the region. Importantly, the Negev is home to multiple minority groups including the Bedouin Arabs, a historically nomadic people. This socioeconomic disadvantage is especially prevalent in the Bedouin community. In 2013, the average salary in Bedouin settlements was only half of the national average [30]. Despite the fact that Bedouin makes up almost a quarter of the residents in the region, there is very little research on the oncologic outcome in this population, especially in breast cancer [31]. The lack of studies on Bedouin women can be attributed to the fact that they face additional barriers to accessing healthcare compared to Jewish women such as gender inequality, poor cancer awareness, and language barrier with healthcare providers [32]. Even though there is a national free breast cancer screening program, the screening rate remains lower in Arab women compared to Jewish women [33]. In this study, we saw that the only cancer-related death occurred in a Bedouin patient due to metastatic disease. The local recurrence rate is not significantly different between Bedouin and Jewish patients after IORT. This result showed that IORT can be potentially advantageous in low socioeconomic groups who cannot afford daily postoperative radiotherapy either due to cost or time constraints. Consistent with a previous study, we found that Bedouin patients are diagnosed at an earlier age with larger tumors than Jewish patients [34]. The nonsignificant difference between the two groups is likely attributed to the small sample size of Bedouin patients which decreases the power of detection. Bedouin patients also had higher rates of receiving additional WBRT than Jewish patients, likely due to more aggressive tumors. Whether those differences are caused by socioeconomic factors or tumor biology, they should be investigated in the future.

The current study has several limitations. First, this is a single-center analysis with a relatively small sample size. Most patients are Jewish, and the number of Bedouin patients is small. A study with a larger sample size will allow for a better comparison between those two groups with increased sensitivity. Nonetheless, the main purpose of this study is to investigate the efficacy and safety of IORT in populations from less-developed regions, and our results showed strong support for the clinical utility of IORT in such a scenario. Second, because the data were retrospectively collected without randomized groups, we cannot directly compare the treatment outcome and toxicity profile between IORT and WBRT. Lastly, the median follow-up is 43.3 months, which is relatively short. Some studies reported a higher rate of local recurrence in the IORT group compared to WBRT only after a longer period of follow-up [35, 36]. This could be affected by many factors including adjuvant chemo and hormonal therapies that suppress the proliferation of leftover cancer cells. In our study, the local recurrence rate is 0.5%, and the distal metastasis rate is 0.9% at a median follow-up of almost 4 years. This result is comparable to reports from other IORT studies. For example, a study from Germany found a low 5-year recurrence rate of 2% which increased to 6.6% at 10 years and 10.1% at 15 years [37]. It is possible in our cohort of patients that the recurrence and metastasis rate will increase as more time passes from the initial BCS with IORT. We will continue follow-up surveillance with our patients in the clinic, and we hope to report long-term data on recurrence and treatment-associated adverse effects.

## 5. Conclusion

Our study showed BCS with IORT achieved excellent prognostic outcomes in suitable patients. It is also safe, with a low rate of tissue toxicity and cosmetic complications. Importantly, IORT reduces treatment-associated burden for patients since it is a one-time irradiation procedure during the initial surgery. This characteristic of IORT is especially advantageous in underdeveloped areas where access to prolonged radiation treatment can be difficult. Future studies should focus on two important areas: providing long-term data on survival outcomes and treatment-associated adverse effects and reporting the clinical utility of IORT in underdeveloped areas and disadvantaged populations.

## Figures and Tables

**Table 1:** Baseline patient demographics and tumor characteristics.

**Patient and tumor characteristics**	**All (** **N** = 211**)**	**Jews (** **N** = 201**)**	**Bedouin (** **N** = 10**)**	**p** **value**
Age (years), mean (SD)	67.5 (6.3)	67.6 (6.4)	65.4 (4.5)	0.17
Tumor type, *n* (%)				**0.001**
IDC	205 (97.1%)	198 (98.5%)	7 (70%)	
ILC	3 (1.4%)	2 (1%)	1 (10%)	
Mucinous carcinoma	3 (1.4%)	1 (0.5%)	2 (20%)	
ER-positive, *n* (%)	211 (100%)	201 (100%)	10 (100%)	1
PR-positive, *n* (%)	199 (94.3%)	190 (94.5%)	9 (90%)	0.45
HER2, *n* (%)				0.31
Positive	11 (5.2%)	11 (5.5%)	0 (0%)	
Negative	170 (80.6%)	163 (81.1%)	7 (70%)	
Unknown	30 (14.2%)	27 (13.4%)	3 (30%)	
Luminal A	159 (75.4%)	153 (76.1%)	6 (60%)	1
Luminal B	1 (0.5%)	1 (0.5%)	0 (0%)	
Tumor stage, *n* (%)				0.65
T1a	13 (6.2%)	13 (6.5%)	0 (0%)	
T1b	73 (34.6%)	69 (34.3%)	4 (4%)	
T1c	114 (54%)	109 (54.2%)	5 (5%)	
T2	11 (5.2%)	10 (5%)	1 (1%)	
Tumor size (cm), mean, (SD)	1.13 (0.44)	1.13 (0.44)	1.21 (0.5)	0.62
Less or equal to 1 cm	99 (46.9%)	95 (47.3%)	4 (40%)	
1–2 cm	105 (49.8%)	99 (49.3%)	6 (60%)	
2–3 cm	7 (3.3%)	7 (3.5%)	0 (0%)	
N stage, *n* (%)				0.6
0	193 (91.5%)	184 (91.5%)	9 (90%)	
1–3	18 (8.5%)	17 (8.5%)	1 (10%)	
ONCOTYPE	61 (28.9%)	57 (28.4%)	4 (40%)	0.479
1–10	12	11	1	
11–15	17	16	1	
16–20	13	13		
21–25	8	7	1	
26–100	11	10	1	

*Note:* Highlighted *p* values are statistically significant.

Abbreviations: ER, estrogen receptor; HER2, human epidermal growth factor receptor 2; IDC, intraductal carcinoma; ILC, intralobular carcinoma; PR, progesterone receptor; SD, standard deviation.

**Table 2:** Treatment modalities and prognostic outcomes between Jew and Bedouin patients.

	**All (** **N** = 211**)**	**Jews** (**N** = 201**)**	**Bedouin** (**N** = 10**)**	**p** **value**
Follow-up months, mean (SD)	43.4 (26.1)	43.8 (25.9)	36.4 (29.9)	0.46
Adjuvant radiotherapy, *n* (%)	27 (12.8%)	23 (11.4%)	4 (40%)	**0.03**
Adjuvant hormonal, *n* (%)	204 (96.7%)	195 (97.5%)	9 (90%)	0.26
Recurrence, *n* (%)	3 (1.4%)	2 (1.5%)	1 (10%)	0.14
Local	1 (0.47%)	1 (0.5%)	0 (0%)	
Metastasis	2 (0.9%)	1 (0.5)	1 (10%)	
Death related to cancer, *n* (%)	1 (0.47%)	0 (0%)	1 (10%)	**0.05**

*Note:* Highlighted *p* values are statistically significant.

Abbreviation: SD, standard deviation.

## Data Availability

Data is available on reasonable request from the corresponding author.
